# Changes in MicroRNA Expression in the Whole Hippocampus and Hippocampal Synaptoneurosome Fraction following Pilocarpine Induced Status Epilepticus

**DOI:** 10.1371/journal.pone.0053464

**Published:** 2013-01-07

**Authors:** Rashmi M. Risbud, Brenda E. Porter

**Affiliations:** 1 Division of Pediatric Neurology, Department of Pediatrics, The Children’s Hospital of Philadelphia, Philadelphia, Pennsylvania, United States of America; 2 Division of Pediatric Neurology, Department of Neurology, The Perelman School of Medicine at the University of Pennsylvania, Philadelphia, Pennsylvania, United States of America; University of South Florida, United States of America

## Abstract

MicroRNAs regulate protein synthesis by binding non-translated regions of mRNAs and suppressing translation and/or increasing mRNA degradation. MicroRNAs play an important role in the nervous system including controlling synaptic plasticity. Their expression is altered in disease states including stroke, head injury and epilepsy. To better understand microRNA expression changes that might contribute to the development of epilepsy, microRNA arrays were performed on rat hippocampus 4 hours, 48 hours and 3 weeks following an episode of pilocarpine induced status epilepticus. Eighty microRNAs increased at one or more of the time points. No microRNAs decreased at 4 hours, and only a few decreased at 3 weeks, but 188 decreased 48 hours after status epilepticus. The large number of microRNAs with altered expression following status epilepticus suggests that microRNA regulation of translation has the potential to contribute to changes in protein expression during epileptogenesis. We carried out a second set of array’s comparing microRNA expression at 48 hours in synaptoneurosome and nuclear fractions of the hippocampus. In control rat hippocampi multiple microRNAs were enriched in the synaptoneurosomal fraction as compared to the nuclear fraction. In contrast, 48 hours after status epilepticus only one microRNA was enriched in the synaptoneurosome fraction. The loss of microRNAs enriched in the synaptoneurosomal fraction implies a dramatic change in translational regulation in synapses 48 hours after status epilepticus.

## Introduction

Epilepsy is a common disorder effecting up to 1% of the population, with one-third of patients having seizures that are refractory to medications. There currently are no treatments aimed at preventing the development of epilepsy. Molecular changes contributing to the development of epilepsy are under intense study in the hopes of identifying therapeutic targets for preventing epilepsy. Inducing a prolonged seizure in rodents, status epilepticus (SE), is a model for studying epileptogenesis; as the animals eventually go on to develop spontaneous seizures. Hopefully, in the future studies of epileptogenesis in rodents can lead to pharmacologic interventions targeting specific pathways for preventing epilepsy in humans.

MicroRNAs regulate protein production by binding to the 3′ end of mRNAs and blocking translation and/or increasing degradation. A microRNA may have hundreds of unique mRNAs targets making altered expression of a single microRNA capable of regulating multiple cellular pathways. Changes have been identified in microRNA expression in multiple neurologic diseases including stroke, head trauma and epilepsy [1], [2], [3], [4], [5], [6], [7], [8]. A recent study has suggested that suppression of microRNA 138 can diminish the severity of kainite induced SE [9]. MicroRNA function can be manipulated by injection of mimics or antagomirs making them potential therapeutic targets for the treatment or prevention of epilepsy.

Using a variety of techniques several labs have shown that microRNAs are present in dendrites and a subset are highly enriched in dendrites [10], [11], [12]. While a few microRNAs present at the synapse have been implicated in regulating synaptic plasticity, the function of most microRNAs in the brain have not been determined [13], [14], [15], [16], [17], [18].

Status epilepticus has been shown to cause destruction and loss of synaptic boutons that to some extent recover once the ongoing seizure activity resolves [19], [20]. Chronically, there are changes in dendrite complexity in the hippocampus of humans and animals with epilepsy [21], [22]. The mechanisms for synaptic bouton and dendrite changes during SE and chronically in epilepsy are not known, though glutamate receptor activation with calcium influx has been proposed as a possible mechanism [23]. How changes in microRNA expression might be contributing to changes in synaptic plasticity or dendrite injury following SE has not been studied.

Here we measure changes in microRNA expression in whole hippocampal samples and in subcellular fractionated samples of the hippocampus following pilocarpine induced SE. There are multiple microRNAs that change following SE and the pool of synaptoneurosome enriched microRNAs diminishes 48 hours after SE.

## Methods

This study was carried out in strict accordance with the recommendations in the Guide for the Care and Use of Laboratory Animals of the National Institutes of Health. The Institutional Animal Care and Use Committee of the Children’s Hospital of Philadelphia approved the experimental protocol.

### Induction of SE

Adult male CD (sprague-dawley) rats from Charles River between 60 to 90 days of age underwent pilocarpine induced SE. All animals received methyl-scopolamine, 1 mg/kg intraperitoneal (IP) 30 minutes prior to the pilocarpine injection to block peripheral cholinergic effects. Status epilepticus was induced with pilocarpine (385 mg/kg, IP), with a half dose given 1 hour later if a Racine stage V, tonic clonic, seizure did not occur [24]. Control rats received, 1/10^th^ dose or 38.5 mg/kg of pilocarpine IP to partially control for pilocarpine effects. The SE rats were monitored for appearance of stage V racine seizures, and received a dose of valium 6 mg/kg IP 1 hour after induction of a stage V seizure. If the animal was still having behavioral seizures 2 hours after the first dose of valium it recieved 3 mg/kg dose of valium every 2 hours as needed until the behavioral seizures resolved. Control animals receive valium doses similar to the SE group to control for the effects of valium.

### Tissue Collection

Rats were sacrificed 4 hours, 48 hours, and 3 weeks after the induction of SE. Rats were anesthetized with isoflurane and hippocampal tissue was dissected out and fast frozen, sonicated and RNA extracted using the mirVANA miRNA isolation kit (Ambion Inc., Austin, Texas, USA). The quality and quantity of RNA are assessed on the Agilent Bioanalyzer for presence of 5, 5.8, 18S bands.

### MicroRNA Array

An Exicon 10.2 array (Exicon,Denmark) was carried out following the companies protocol. Two micrograms of total hippocampal RNA from 4 hour, 48 hour and 3 week control and SE treated animals was labeled with Cy3 or Cy5 aminoallyl tailing to make the fluorescent probes using the Exicon labeling kit. We took a dye swap approach, a control-Cy3 and seizure-Cy5 sample was probed on an array then the same samples with the dyes switched, seizure-Cy3 and control-Cy5 were probed on an array [25], [26]. This was done for control and SE animals at each time point. Similarly, for the synaptoneurosome and nuclear fraction array studies, a nuclear-Cy3 and a synaptoneurosome-Cy5 sample was probed on an array then the same samples with the dyes switched, synaptoneurosome-Cy5 and a nuclear-Cy3 was probed on an array. A t-test was performed for microRNAs expressed above background.

### Preparation of Synaptoneurosome Fraction and Nuclear Pellet

Hippocampal synaptoneurosomes were prepared as originally described [27], [28]. All steps were conducted on ice or 4°C, and all solutions were made using diethylpyrocarbonate-treated and nuclease-free water. Briefly, hippocampi were gently homogenized at 4°C in 10 times volume of isolation media (0.32 M sucrose, 10 mM Tris-HCl, 1 mM EDTA). After a low speed centrifugation step to remove cell bodies, the resulting supernatant was centrifuged at 12,500 RPM for 20 min using a Beckman JA-17 rotor. The resulting pellet was gently suspended in a small volume of isolation media and then brought with 12% Ficoll in a total volume of 5.5 ml. After layering 3 mL of 7% Ficoll over this solution, followed by 3.3 mL of isolation media, the samples underwent ultracentrifugation at 27,000 RPM for 30 min using a Beckman SW-41ti rotor. Synaptoneurosomes were isolated at the 7%/12% interface and washed four times (10 mL per wash) in isolation media. One aliquot of this material was analyzed for protein using the BCA protein assay kit (Pierce, Rockford, IL.). A second aliquot was diluted in 2X SDS sample buffer. We showed that these synaptosoneurosomes contain virtually no histone H3, suggesting that they are relatively free of cell bodies [29].

### Real Time-PCR

Specific stem loop miRNA primers from the Taqman MicroRNA Assays and reagents from the Reverse Transcription Kit (Applied Biosystems) were used to transcribe miR 124, 103, 30c, 128a, 138, 21, 21*, Let 7b and 4.5 s. Concentrations of RNA and cDNA were measured using a spectrophotometer (ND 1000, Thermo Fischer Scientific Inc, Wilmington, DE). Reactions were carried out in 384-well plates with 5 µl of the Taqman Universal PCR Master Mix (Applied Biosystems, Branchburg, NJ), 0.5 µl of the probe/primer mix for either the gene of interest, 2.5 µl of ddH2O, and 2 µl of sample DNA per well. Concentrations of DNA were diluted so that approximately 1000ηg–1100ηg of sample DNA were added to each well. Each sample was run in two sets of triplicates, the No AmpErase UNG (Applied Biosystems) enzyme was used (Applied Biosystems) with one set with the probe for the gene of interest and one for 4.5S ribonuclear small RNA. A standard curve for each probe was included on each plate using cDNA from the cortex of a control rat. The real-time PCR assays were carried out on a SDS 7900HT model thermocycler (Applied Biosystems). The real-time PCR settings were 50°C for 2 minutes, 95°C for 10 minutes, and then 40 cycles of 95°C for 1 minutes and 60°C for 1 minute. The housekeeping genes, 4.5 s for the hippocampus samples did not vary across treatment group for any of the time points.

Reverse transcription using random hexamers was performed using a Superscript II Reverse Transcription kit for mRNAs (Invitrogen, Carlsbad, California, USA). Reactions were carried out in 384-well plates with 5 µl of the Taqman Universal PCR Master Mix (Applied Biosystems, Branchburg, NJ), 0.5 µl of the probe/primer mix for either the gene of interest HDAC3 (Rn00584926_m1), GAPDH (Rn01775763_g1), CAMK2α (Rn00563883_m1), DLGAP2 also known as PSD95/SAP90, (Rn00588099_m1), GRIA2 also known as GLUR2 (Rn00568514_m1), MTAP2 also known as MAP2 (Rn00565046_m1) 2.5 ul of ddH2O, and 2 µl of sample DNA per well. Concentrations of cDNA were diluted so that approximately 1000 ηg–1100ηg of sample cDNA were added to each well. Each sample was run in two sets of triplicates, one set with the probe for the gene of interest and one for PPIA, cyclophilin. A standard curve for each probe was included on each plate using cDNA from the cortex of a control rat.

## Results

**Figure 1:**
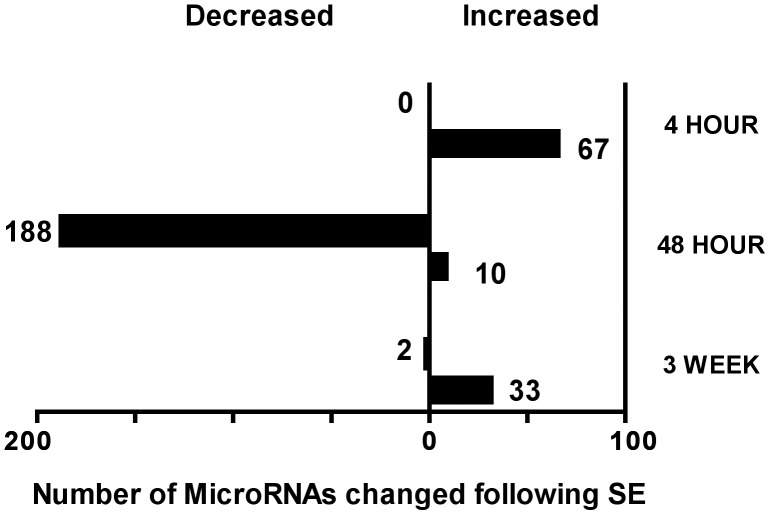
Total number of hippocampal microRNAs that are increased or decreased after SE at each time point. There are a large number of microRNAs that specifically decreased at 48 hours after SE. The specific microRNAs that change are listed on Tables 1, 2 and 3. We used a P value of 0.05 on a t-test to identify microRNAs that change.

Rats were sacrificed at 4 hours, 48 hours and 3 weeks, following pilocarpine induced SE, encompassing the time period over which most of the rats will develop chronic epilepsy. Typically, using this model rats developed epilepsy by 8–15 days after SE [10], [30], [31]. To identify microRNAs that change after SE, MicroRNA array analysis on whole hippocampus was performed. Table 1 displays microRNAs that increased following SE; Table 2 displays microRNAs that increased at one time point and decreased at another time point; and Table 3 are microRNAs that only decreased following SE. Four hours after SE using a t-test cutoff <0.05, there is an increase in 67 microRNAs and none that decreased (Figure 1 and Tables 1, and 2). Forty-eight hours after SE 10 microRNAs increased, and 188 decreased (Figure 1 and Table 2 and 3). By three weeks after SE there were 33 microRNAs that increased, and 3 that decreased, (Figure 1 Table 1, 2, and 3).

**Table 1:** MicroRNA levels that increased following SE.

	4 HOUR DATA		48 HOUR DATA		3 WEEK DATA	
MicroRNA Name	SZ/CNTL	T-Test	SZ/CNTL	T-Test	SZ/CNTL	T-Test
hsa-miR-21	0.996	0.979	**2.821**	**0.010**	**1.941**	**0.018**
hsa-miR-132	**1.431**	**0.007**	1.232	0.494	1.512	0.220
hsa-miR-142-3p	1.107	0.387	1.483	0.091	**2.874**	**0.019**
hsa-miR-155	0.983	0.970	**1.976**	**0.007**	**1.326**	**0.032**
hsa-miR-184	**1.333**	**0.021**	1.005	0.971	1.121	0.156
hsa-miR-19a	0.976	0.806	0.861	0.481	**1.757**	**0.042**
hsa-miR-19b	0.990	0.937	0.833	0.472	**1.716**	**0.052**
hsa-miR-214	**1.574**	**0.022**	1.105	0.372	1.206	0.266
hsa-miR-516b	**1.572**	**0.018**	1.176	0.429	1.001	0.995
mmu-miR-470	**2.042**	**0.001**	2.839	0.254	0.866	0.286
hsa-miR-518c*	**1.820**	**0.003**	1.918	0.095	1.138	0.139
hsa-miR-519e*	**1.408**	**0.019**	1.244	0.238	1.050	0.562
hsa-miR-519c-5p	**1.349**	**0.030**	1.251	0.201	1.077	0.298
mmu-miR-503	**1.380**	**0.014**	1.004	0.974	1.138	0.229
mghv-miR-M1-4	**1.511**	**0.005**	1.202	0.356	1.083	0.224
hsa-miR-583	**2.837**	**0.002**	3.084	0.096	**1.224**	**0.035**
ebv-miR-BART8*	**3.309**	**0.004**	5.305	0.105	**1.745**	**0.004**
hsa-miR-637	**5.037**	**0.010**	6.385	0.264	1.157	0.266
mghv-miR-M1-8	**1.430**	**0.044**	**1.201**	**0.047**	1.075	0.260
mmu-miR-685	1.401	0.100	**5.099**	**0.008**	**1.374**	**0.023**
kshv-miR-K12-6-3p	**4.015**	**0.013**	5.102	0.255	1.009	0.969
sv40-miR-S1-5p	**2.274**	**0.001**	**5.171**	**0.042**	**1.903**	**0.014**
mmu-miR-713	**1.466**	**0.040**	0.904	0.401	0.974	0.745
hsa-miR-658	**2.650**	**0.004**	2.848	0.139	1.186	0.106
kshv-miR-K12-8	**1.484**	**0.019**	1.274	0.220	**1.161**	**0.026**
mmu-miR-467b*	**1.461**	**0.052**	0.911	0.280	0.907	0.583
hsa-miR-132*	**1.438**	**0.036**	1.030	0.924	1.480	0.174
hsa-miR-934	**1.485**	**0.054**	1.096	0.740	1.074	0.258
hsa-miR-125b-1*	**1.516**	**0.008**	1.082	0.776	1.184	0.067
hsa-miR-193a-5p	**5.438**	**0.005**	12.986	0.145	**1.587**	**0.010**
mmu-miR-21*	**5.098**	**0.008**	7.390	0.253	1.331	0.125
hsa-miR-185*	**2.034**	**0.005**	1.998	0.370	1.002	0.983
miRPlus_17952	1.371	0.364	2.951	0.198	**1.296**	**0.028**
hsa-miR-183*	**2.065**	**0.005**	1.486	0.309	1.131	0.219
hsa_SNORD3@	1.547	0.072	**1.492**	**0.023**	1.069	0.399
hsa_SNORD2	1.056	0.639	1.068	0.636	**1.419**	**0.008**
hsa-miR-142-5p	1.106	0.451	1.359	0.172	**2.397**	**0.020**
miRPlus_27560	**10.615**	**0.016**	28.150	0.215	1.721	0.093
hsa-miR-423-5p	**1.663**	**0.006**	1.220	0.146	1.095	0.225
mmu-miR-711	**2.609**	**0.014**	8.477	0.119	1.389	0.081
hsa-miR-765	**2.222**	**0.005**	1.910	0.064	1.111	0.384
miRPlus_28431	**1.856**	**0.009**	1.177	0.634	0.990	0.895
mmu-miR-675-5p	**1.564**	**0.007**	2.493	0.100	**1.205**	**0.013**
hsa-miR-498	**2.833**	**0.001**	11.875	0.065	**1.406**	**0.044**
mmu-miR-883a-5p	**1.310**	**0.036**	0.952	0.609	1.092	0.366
hsa-miR-149*	**1.605**	**0.024**	4.634	0.058	1.145	0.091
miRPlus_42487	**3.349**	**0.003**	6.613	0.137	**1.512**	**0.008**
hsa-miR-516a-5p	**7.348**	**0.014**	7.293	0.216	**1.478**	**0.046**
mmu-miR-881*	**2.081**	**0.005**	**2.083**	**0.053**	1.011	0.922
hsa-miR-212	**1.873**	**0.001**	1.536	0.176	1.519	0.223
hsa-miR-885-3p	**1.924**	**0.003**	2.909	0.184	1.065	0.606
hsa-miR-20b	1.016	0.896	0.919	0.680	**1.816**	**0.039**
hsa-miR-106a	0.982	0.895	1.077	0.687	**1.888**	**0.035**
hsa-miR-20a	0.989	0.938	1.088	0.713	**1.783**	**0.040**
hsa-miR-17	0.983	0.895	1.040	0.834	**1.912**	**0.037**
hsa-miR-483-5p	**2.130**	**0.003**	**3.004**	**0.016**	1.084	0.706
hsa-miR-642	**1.999**	**0.004**	1.980	0.077	0.975	0.764
hsa-miR-485-3p	**1.522**	**0.028**	1.391	0.155	0.956	0.633
hsa-miR-625*	**1.755**	**0.009**	1.866	0.059	0.967	0.620
hsa-miR-30c-2*	**1.726**	**0.028**	1.436	0.362	1.077	0.215
hsa-miR-423-3p	1.236	0.056	0.919	0.598	**1.212**	**0.022**
hsa-miR-371-5p	**3.251**	**0.004**	10.844	0.094	**2.163**	**0.004**
miRPlus_42745	**3.521**	**0.003**	5.459	0.152	1.415	0.070
hsa-miR-187*	**2.857**	**0.001**	1.294	0.663	1.027	0.810
hsa-miR-921	**1.525**	**0.026**	1.211	0.526	1.089	0.321
miRPlus_42793	**1.885**	**0.007**	**2.026**	**0.045**	1.060	0.409
mmu-miR-300*	**1.500**	**0.035**	1.205	0.556	1.082	0.440
hsa-miR-638	**3.270**	**0.002**	4.277	0.172	1.214	0.146
mmu-miR-20b	0.986	0.900	1.415	0.121	**1.814**	**0.040**
mmu-miR-882	**2.812**	**0.001**	4.166	0.056	1.085	0.322
ebv-miR-BHRF1-1	**1.391**	**0.032**	1.007	0.959	1.018	0.786
hsa-miR-25*	**2.070**	**0.008**	2.453	0.180	**1.197**	**0.020**
hsa-miR-193b*	**4.354**	**0.014**	5.330	0.155	1.072	0.602
hsa-miR-550	1.084	0.542	0.765	0.054	**1.267**	**0.024**
hsa-miR-300	**1.453**	**0.011**	**1.351**	**0.010**	0.905	0.470
mmu-miR-763	**1.299**	**0.027**	0.686	0.084	0.958	0.421
mmu-miR-294*	**1.478**	**0.007**	1.441	0.158	1.090	0.235
hsa-miR-518a-5p	**1.405**	**0.010**	0.872	0.360	1.098	0.363
hsa-miR-551a	**1.756**	**0.003**	1.358	0.420	0.889	0.417
hsa-miR-198	**1.550**	**0.027**	1.025	0.900	0.804	0.137
rno-miR-204*	**1.305**	**0.049**	0.859	0.564	1.013	0.897
hsa-miR-34a	1.124	0.419	0.634	0.133	**1.282**	**0.041**

SZ-seizure, CNTL-control, Bolded ratios have a t-test P value<0.05.

**Table 2:** MicroRNA levels that decreased and increased following SE.

	4 HOUR DATA		48 HOUR DATA		3 WEEK DATA	
MicroRNA Name	SZ/CNTL	T-Test	SZ/CNTL	T-Test	SZ/CNTL	T-Test
hsa-miR-146b-5p	0.994	0.970	**0.671**	**0.051**	**1.516**	**0.048**
hsa-miR-146a	1.125	0.251	**0.664**	**0.027**	**2.326**	**0.015**
miRPlus_28302	1.136	0.203	**0.759**	**0.011**	**1.485**	**0.001**
hsa-miR-574-3p	**1.333**	**0.010**	**0.777**	**0.014**	0.882	0.431
hsa-miR-422a	1.099	0.381	**0.599**	**0.013**	**1.394**	**0.035**
mmu-miR-465b-5p	1.221	0.151	**0.658**	**0.011**	**1.511**	**0.012**
miRPlus_42530	**1.239**	**0.015**	**0.840**	**0.040**	0.975	0.736
hsa-miR-298	**1.392**	**0.029**	**0.725**	**0.046**	0.956	0.775

SZ-seizure, CNTL-control, Bolded ratios have a t-test P value<0.05.

**Table 3:** MicroRNA levels that decreased following SE.

	4 HOUR DATA		48 HOUR DATA		3 WEEK DATA	
MicroRNA Name	SZ/CNTL	T-Test	SZ/CNTL	T-Test	SZ/CNTL	T-Test
hsa-miR-9	0.936	0.478	**0.474**	**0.005**	0.895	0.594
hsa-miR-126	0.994	0.956	**0.736**	**0.049**	1.177	0.445
mmu-let-7i	0.944	0.424	**0.539**	**0.016**	1.052	0.755
hsa-miR-130a	1.042	0.689	**0.638**	**0.007**	1.229	0.276
mmu-let-7d	0.921	0.420	**0.502**	**0.012**	1.014	0.939
hsa-miR-103	0.957	0.693	**0.527**	**0.008**	0.973	0.876
hsa-miR-107	0.981	0.815	**0.508**	**0.010**	1.053	0.762
hsa-miR-125a-5p	0.941	0.619	**0.532**	**0.014**	0.894	0.554
hsa-miR-137	1.033	0.634	**0.416**	**0.013**	0.848	0.460
hsa-miR-148a	1.022	0.752	**0.512**	**0.040**	1.044	0.768
hsa-miR-154	0.963	0.687	**0.530**	**0.015**	0.921	0.629
hsa-miR-181b	0.974	0.862	**0.478**	**0.050**	1.095	0.573
hsa-miR-191	0.964	0.781	**0.617**	**0.033**	1.087	0.560
hsa-miR-181a*	1.063	0.704	**0.597**	**0.019**	0.957	0.695
hsa-miR-218	0.927	0.473	**0.454**	**0.026**	0.778	0.343
hsa-miR-22	0.936	0.628	**0.620**	**0.026**	1.031	0.827
hsa-miR-221	0.858	0.379	**0.509**	**0.002**	0.907	0.654
hsa-miR-222	0.893	0.530	**0.526**	**0.022**	0.864	0.513
hsa-miR-26a	0.941	0.590	**0.531**	**0.021**	0.949	0.782
hsa-miR-299-5p	1.176	0.215	0.769	0.208	**0.793**	**0.023**
hsa-miR-29b	0.983	0.814	**0.453**	**0.012**	0.838	0.422
hsa-miR-29c	0.997	0.964	**0.463**	**0.027**	0.873	0.511
hsa-miR-30a	0.991	0.929	**0.519**	**0.023**	1.011	0.948
hsa-miR-376b	1.035	0.793	**0.444**	**0.010**	0.884	0.521
hsa-miR-377	1.136	0.270	**0.665**	**0.008**	0.986	0.902
hsa-miR-382	0.968	0.800	**0.580**	**0.012**	0.902	0.479
hsa-miR-378	1.000	0.999	**0.652**	**0.033**	1.385	0.094
hsa-miR-503	1.202	0.100	**0.775**	**0.023**	1.040	0.691
hsa-miR-519d	1.050	0.249	**0.536**	**0.021**	1.087	0.449
hsa-miR-519e	0.976	0.769	**0.626**	**0.027**	0.982	0.821
hsa-miR-98	0.916	0.358	**0.369**	**0.007**	1.007	0.968
hsa-miR-99b	0.939	0.656	**0.582**	**0.043**	1.055	0.755
mmu-miR-207	1.049	0.506	**0.482**	**0.003**	1.003	0.971
mmu-miR-300	1.044	0.623	**0.635**	**0.011**	0.916	0.619
hsa-miR-30e*	0.979	0.857	**0.538**	**0.018**	0.971	0.868
mmu-miR-329	0.996	0.974	**0.566**	**0.024**	0.912	0.642
mmu-miR-345-5p	1.030	0.776	**0.664**	**0.025**	0.941	0.572
mmu-miR-380-3p	0.986	0.882	**0.665**	**0.011**	0.857	0.394
mmu-miR-433*	1.134	0.191	**0.620**	**0.000**	1.108	0.360
mmu-miR-434-3p	1.034	0.757	**0.592**	**0.024**	0.815	0.332
mmu-miR-434-5p	1.065	0.592	**0.554**	**0.023**	0.840	0.381
hsa-miR-151-5p	0.973	0.815	**0.603**	**0.023**	1.028	0.833
rno-miR-333	1.005	0.979	**0.663**	**0.008**	1.064	0.666
rno-miR-336	1.161	0.250	**0.467**	**0.010**	0.899	0.302
rno-miR-352	0.916	0.365	**0.501**	**0.016**	0.919	0.683
rno-miR-450a	0.997	0.978	**0.613**	**0.039**	1.063	0.758
hsa-miR-7-1*	0.966	0.777	**0.500**	**0.012**	**0.713**	**0.027**
U6-snRNA-1	1.082	0.742	**0.774**	**0.042**	1.143	0.071
hsa-miR-138	0.920	0.578	**0.502**	**0.004**	0.787	0.318
hsa-miR-301a	0.945	0.603	**0.564**	**0.010**	0.984	0.940
hsa-miR-369-5p	1.048	0.674	**0.573**	**0.026**	0.824	0.340
hsa-miR-195	0.940	0.297	**0.472**	**0.004**	1.103	0.637
mmu-miR-322	1.020	0.868	**0.544**	**0.015**	0.895	0.544
hsa-miR-10a	1.116	0.171	**0.518**	**0.005**	0.966	0.707
mmu-miR-376a*	1.103	0.386	**0.611**	**0.001**	0.859	0.431
hsa-miR-539	0.997	0.977	**0.564**	**0.027**	0.829	0.362
hsa-miR-487b	1.010	0.869	**0.513**	**0.011**	0.990	0.935
mmu-miR-541	0.987	0.923	**0.638**	**0.019**	0.936	0.691
hsa-miR-29c*	0.976	0.838	**0.507**	**0.008**	0.792	0.172
hsa-miR-361-5p	1.025	0.833	**0.598**	**0.027**	1.001	0.997
hsa-miR-374b	0.985	0.853	**0.521**	**0.018**	1.158	0.253
hsa-miR-381	1.089	0.440	**0.735**	**0.047**	0.951	0.749
rno-miR-664	1.057	0.622	**0.687**	**0.015**	0.988	0.889
hsa-miR-124	1.005	0.939	**0.546**	**0.013**	0.838	0.350
hsa-miR-411	1.000	0.997	**0.566**	**0.027**	0.846	0.380
hsa-miR-30b	1.003	0.982	**0.558**	**0.042**	0.941	0.752
hsa-miR-629*	1.080	0.404	**0.622**	**0.002**	1.274	0.086
mmu-miR-691	1.089	0.578	**0.663**	**0.022**	1.012	0.933
hsa-miR-92b	1.020	0.796	**0.721**	**0.030**	1.106	0.279
hsa-let-7a	0.923	0.436	**0.495**	**0.011**	0.981	0.917
hsa-let-7d	0.934	0.370	**0.428**	**0.003**	0.997	0.985
hsa-let-7f	0.936	0.452	**0.419**	**0.013**	1.008	0.969
hsa-let-7a*	0.948	0.539	**0.514**	**0.014**	1.009	0.956
miRPlus_17891	1.116	0.290	**0.617**	**0.044**	1.018	0.851
hsa-miR-99b*	1.147	0.096	**0.591**	**0.022**	1.055	0.393
hsa-miR-491-3p	0.993	0.938	**0.468**	**0.007**	1.022	0.827
hsa-miR-186	0.968	0.793	**0.608**	**0.016**	0.939	0.682
hsa-miR-450a	0.962	0.787	**0.581**	**0.022**	1.093	0.695
hsa-let-7c	0.933	0.640	**0.557**	**0.028**	1.076	0.674
hsa-let-7i	0.965	0.641	**0.563**	**0.017**	1.075	0.682
hsa-miR-100	0.945	0.702	**0.527**	**0.036**	1.136	0.455
hsa-miR-148b	0.952	0.690	**0.580**	**0.023**	1.010	0.960
hsa-miR-30a*	1.082	0.532	**0.484**	**0.004**	1.026	0.893
hsa-let-7g	0.956	0.396	**0.429**	**0.005**	0.913	0.625
mmu-miR-742	1.051	0.605	**0.557**	**0.013**	1.062	0.366
hsa-miR-374a	1.001	0.991	**0.604**	**0.048**	1.161	0.302
hsa-miR-190	1.012	0.929	**0.507**	**0.026**	1.001	0.994
hsa-miR-139-5p	1.080	0.303	**0.422**	**0.022**	0.719	0.212
hsa-miR-363*	1.076	0.552	**0.542**	**0.038**	1.017	0.891
hsa-miR-744	1.051	0.691	**0.583**	**0.029**	0.909	0.600
hsa-miR-891a	1.097	0.699	**0.750**	**0.034**	1.166	0.155
hsa-miR-30e	0.955	0.547	**0.486**	**0.008**	0.957	0.818
mmu-miR-872	1.024	0.806	**0.564**	**0.045**	1.041	0.801
mmu-miR-741	1.104	0.305	**0.498**	**0.001**	0.864	0.156
mmu-miR-667	1.095	0.327	**0.592**	**0.005**	1.006	0.962
hsa-let-7b	0.940	0.485	**0.468**	**0.011**	1.123	0.526
hsa-miR-9*	0.969	0.758	**0.611**	**0.019**	0.944	0.742
hsa-miR-768-5p	1.125	0.154	**0.525**	**0.016**	1.090	0.381
hsa-miR-340	1.058	0.457	**0.622**	**0.024**	1.170	0.103
hsa-miR-125b	1.000	0.997	**0.564**	**0.016**	1.077	0.652
mmu-miR-804	1.150	0.273	**0.539**	**0.038**	1.127	0.187
mmu-miR-384-3p	0.948	0.553	**0.523**	**0.018**	0.814	0.384
hsa-miR-342-3p	1.009	0.929	**0.603**	**0.025**	1.189	0.298
mmu-let-7f	0.930	0.557	**0.564**	**0.019**	1.016	0.933
hsa-miR-126*	1.031	0.725	**0.600**	**0.011**	1.230	0.315
hsa-miR-505	1.076	0.381	**0.554**	**0.044**	0.894	0.326
mmu-miR-382*	1.212	0.255	**0.714**	**0.009**	1.099	0.454
mmu-miR-376c*	1.138	0.201	**0.487**	**0.049**	0.802	0.083
mmu-miR-598	0.938	0.522	**0.490**	**0.009**	0.880	0.579
hsa-let-7e	1.004	0.971	**0.634**	**0.035**	1.019	0.901
mmu-let-7g	0.936	0.457	**0.513**	**0.010**	0.985	0.934
hsa-miR-485-5p	1.052	0.735	**0.575**	**0.014**	0.825	0.191
hsa-miR-181c	1.089	0.247	**0.580**	**0.019**	1.016	0.936
hsa-miR-136*	1.004	0.963	**0.537**	**0.051**	0.868	0.518
hsa-miR-22*	0.996	0.974	**0.617**	**0.024**	1.033	0.852
hsa-miR-933	1.070	0.354	**0.544**	**0.014**	1.045	0.557
hsa-miR-494	1.090	0.587	**0.600**	**0.007**	1.148	0.265
hsa-miR-589	1.084	0.423	**0.502**	**0.025**	1.012	0.876
hsa-miR-379*	1.071	0.554	**0.585**	**0.012**	0.834	0.368
hsa-miR-26b	0.940	0.617	**0.539**	**0.024**	0.945	0.769
hsa-miR-384	1.029	0.724	**0.494**	**0.045**	0.803	0.201
hsa-miR-634	1.182	0.108	**0.681**	**0.012**	1.013	0.865
mmu-miR-503*	1.095	0.260	**0.641**	**0.028**	1.020	0.818
hsa-miR-376c	1.033	0.709	**0.493**	**0.019**	0.937	0.612
hsa-miR-140-3p	1.024	0.833	**0.635**	**0.047**	1.216	0.217
hsa-miR-145	0.972	0.685	**0.571**	**0.015**	1.605	0.289
mmu-let-7c-1*	1.115	0.419	**0.614**	**0.047**	1.037	0.605
hsa-miR-505	1.042	0.756	**0.604**	**0.007**	1.012	0.911
hsa-miR-337-3p	1.026	0.722	**0.538**	**0.004**	0.947	0.642
hsa-miR-328	1.157	0.093	**0.703**	**0.046**	0.865	0.215
hsa-miR-495	1.039	0.701	**0.596**	**0.026**	0.846	0.393
hsa-miR-136*	1.039	0.711	**0.571**	**0.042**	0.931	0.724
mmu-miR-325	1.026	0.830	**0.650**	**0.027**	0.954	0.819
hsa-miR-99a	0.954	0.697	**0.484**	**0.017**	1.163	0.380
hsa-miR-887	0.978	0.759	**0.503**	**0.009**	1.010	0.924
hsa-miR-33a	0.982	0.838	**0.479**	**0.041**	0.750	0.350
hsa-miR-34b	0.989	0.879	**0.470**	**0.015**	0.983	0.863
hsa-miR-668	1.138	0.172	**0.567**	**0.020**	1.042	0.713
mmu-miR-138	0.941	0.691	**0.479**	**0.002**	0.763	0.292
hsa-let-7e*	1.083	0.540	**0.650**	**0.040**	0.858	0.140
mmu-miR-872*	1.117	0.165	**0.661**	**0.020**	1.061	0.528
hsa-miR-665	1.089	0.601	**0.695**	**0.039**	0.993	0.965
rno-miR-347	1.092	0.378	**0.503**	**0.005**	1.089	0.270
mmu-miR-125b*	1.089	0.343	**0.569**	**0.009**	0.971	0.798
hsa-miR-411*	1.065	0.572	**0.602**	**0.020**	0.907	0.622
mmu-miR-337-3p	0.988	0.916	**0.677**	**0.016**	1.109	0.489
hsa-miR-29b-2*	1.053	0.643	**0.548**	**0.024**	0.809	0.168
hsa-miR-582-5p	0.946	0.676	**0.465**	**0.048**	0.788	0.303
hsa-miR-150	1.131	0.321	**0.719**	**0.048**	0.909	0.518
hsa-miR-149	1.066	0.584	**0.514**	**0.005**	0.788	0.169
hsa-miR-218-2*	1.024	0.822	**0.459**	**0.002**	0.920	0.386
hsa-miR-597	1.091	0.361	**0.479**	**0.004**	1.037	0.728
hsa-miR-888*	1.047	0.475	**0.515**	**0.006**	1.119	0.260
hsa-miR-127-3p	1.010	0.941	**0.563**	**0.025**	0.904	0.514
hsa-miR-28-5p	0.981	0.843	**0.583**	**0.024**	1.115	0.462
hsa-miR-181d	1.028	0.681	**0.433**	**0.016**	1.047	0.754
mmu-miR-384-5p	1.001	0.990	**0.578**	**0.014**	0.848	0.352
hsa-miR-497	0.921	0.227	**0.463**	**0.003**	0.897	0.591
hsa-miR-433	0.997	0.982	**0.665**	**0.018**	1.003	0.978
miRPlus_42856	1.089	0.510	**0.766**	**0.050**	1.144	0.170
rno-miR-743b	1.132	0.186	**0.545**	**0.004**	1.054	0.488
hsa-miR-181a	0.992	0.942	**0.510**	**0.044**	1.023	0.895
mmu-miR-337-5p	0.994	0.965	**0.603**	**0.029**	0.939	0.645
hsa-miR-138-1*	1.035	0.701	**0.515**	**0.002**	1.001	0.995
hsa-miR-331-3p	0.937	0.534	**0.544**	**0.014**	0.986	0.919
mmu-miR-875-3p	1.098	0.569	**0.701**	**0.016**	1.014	0.912
hsa-miR-379*	1.152	0.160	**0.582**	**0.039**	0.859	0.322
mmu-miR-467e*	1.061	0.814	**0.760**	**0.047**	0.972	0.856
hsa-miR-124*	1.103	0.473	**0.653**	**0.041**	0.817	0.123
hsa-miR-185	0.996	0.977	**0.596**	**0.020**	0.958	0.807
mmu-miR-327	0.780	0.329	**0.419**	**0.035**	0.905	0.542
hsa-miR-551b	1.040	0.673	**0.522**	**0.034**	0.792	0.241
hsa-miR-29a*	1.032	0.793	**0.522**	**0.022**	0.951	0.777
hsa-miR-30c	0.982	0.877	**0.511**	**0.009**	0.925	0.695
hsa-miR-409-5p	1.047	0.724	**0.468**	**0.011**	0.929	0.632
mmu-miR-582-3p	1.053	0.763	**0.657**	**0.023**	1.128	0.311
hsa-miR-134	1.076	0.514	**0.599**	**0.048**	1.013	0.901
ebv-miR-BART17-5p	1.248	0.068	**0.803**	**0.049**	0.952	0.610
mmu-miR-101b	0.949	0.545	**0.549**	**0.042**	1.086	0.650
hsa-miR-323-3p	0.975	0.809	**0.558**	**0.018**	0.875	0.535
rno-miR-742	1.066	0.619	**0.683**	**0.023**	1.099	0.352
hsa-miR-7-2*	1.227	0.220	**0.680**	**0.049**	0.848	0.107
hsa-miR-615-3p	1.249	0.058	0.894	0.497	**0.890**	**0.039**

SZ-seizure, CNTL-control, Bolded decreased ratios have a t-test P value<0.05.

**Figure 2:**
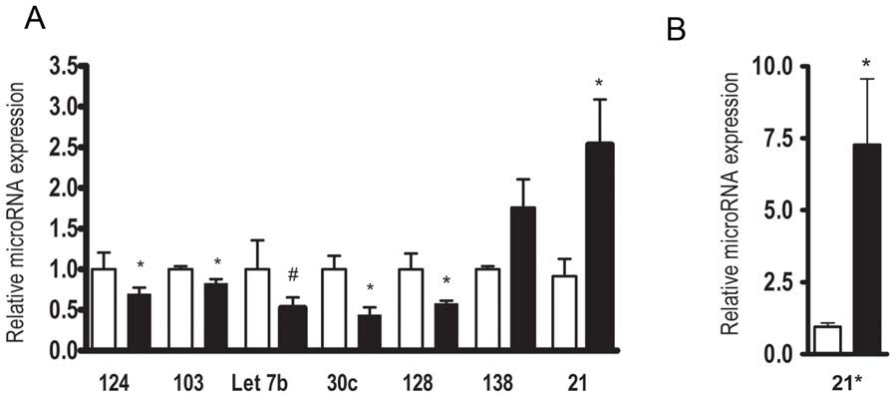
Real time PCR confirmed changes in 6 of 8 microRNAs identified as either decreased or increased on the hippocampal microRNA arrays following SE. P*<0.05 T-test, control N = 8 to 9 and SE N = 7 to 9 samples per group.

To determine if the microRNA changes identified on the array could be validated using a second technique, real-time PCR was carried out on hippocampus samples at 48 hours after SE (Figure 2). Of eight microRNAs tested six of the eight had a difference in the direction identified on the array. Let-7b trended downward similar to the array, but was not significantly lower. MiR138 had decreased on the array but no difference was found in its level by real time PCR, with a trend toward an increased level.

**Figure 3:**
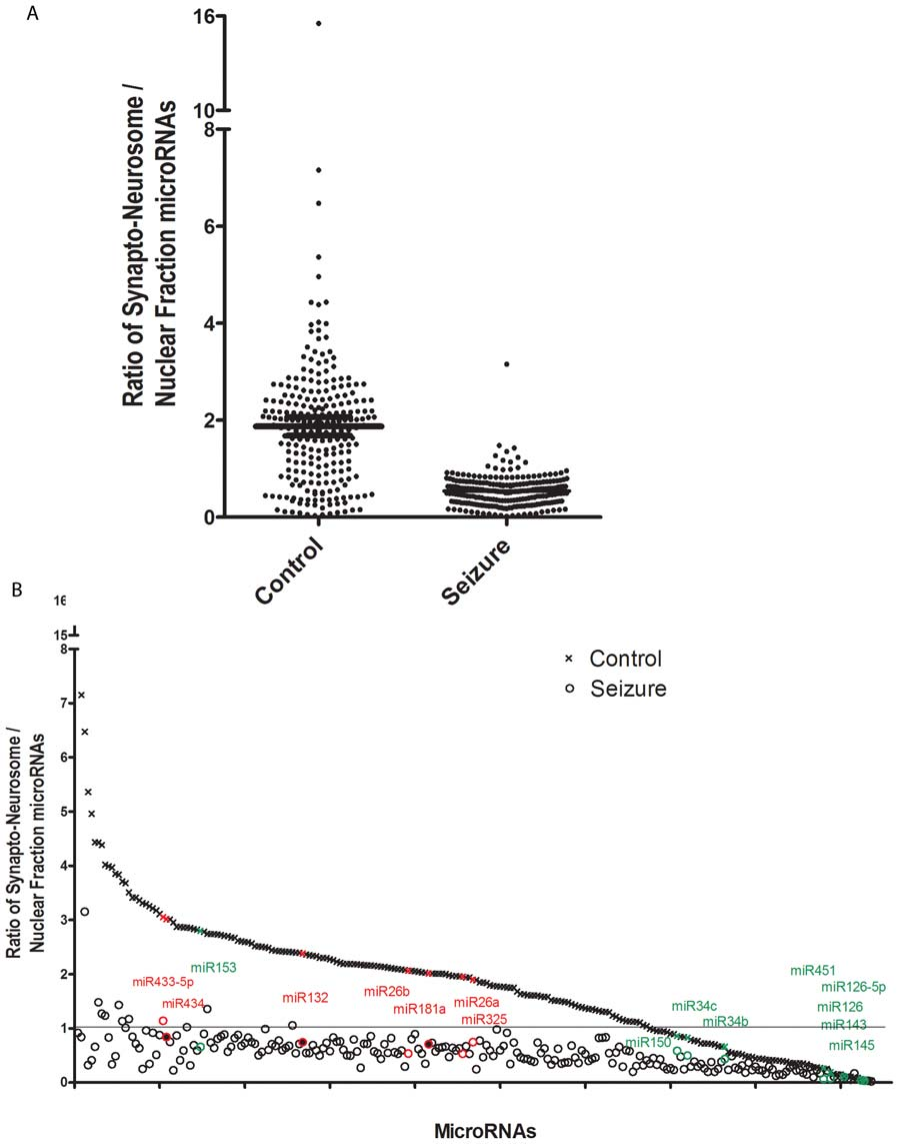
There is a decrease in the ratio of synaptoneurosome/nuclear microRNA fractions in the hippocampus following SE. A) A dot plot of the synaptoneurosome/nuclear ratio of all of the microRNAs expressed above background in at least four arrays from either nuclear or synaptoneurosome fractions. MicroRNAs in the hippocampus of control animals and 48 hours after SE are plotted with the mean and standard error shown by black lines. B) The synaptoneurosome/nuclear ratio of all of the microRNAs expressed above background are plotted left to right, based on the microRNAs that are most highly enriched to the least in the synaptoneurosome fraction in the control hippocampus. X represents the synaptoneurosome/nuclear microRNA ratio of the control samples, and directly below each X the 0 is the same microRNAs synaptoneurosome/nuclear ratio of the SE samples. Specific microRNAs and their synaptoneurosome/nuclear microRNA ratio are shown in Table 4. We have highlighted in red a subset of microRNAs that have previously been shown to be enriched in dendrites or synaptoneurosomes and highlighted in green a subset of microRNAs that have previously been shown to be expressed at low levels in dendrites or synaptoneurosomes [10], [11], [31].

Prior studies found that microRNAs are prominently expressed in dendrites in vitro and a subset of microRNAs are enriched in synaptoneurosomes of the cortex and hippocampus [10], [11], [32]. Subcellular fractionation of the hippocampus was performed to determine if there was a redistribution of microRNAs from the synaptoneurosome pool following SE. Multiple microRNAs were found to be enriched in the synaptoneurosome fraction in the control animals, i.e. a synaptoneurosome/nuclear ratio >1, see Figure 3A. Forty-eight hours after SE microRNAs were not enriched in the synaptoneurosome fraction. Table 4 lists the synaptoneurosome/nuclear ratio of each microRNA from the most highly synaptoneurosome enriched to the lowest, in control animals and 48 hours after SE. In Figure 3B the control synaptoneurosome/nuclear ratios, highest to lowest, are plotted with an x; the o directly below the X is the synaptoneurosome/nuclear ratio of the same microRNA 48 hours after SE. There is a dramatic reduction in the highly enriched synaptoneurosome/nuclear microRNAs 48 hours after SE.

**Table 4:** Ratio of hippocampal synaptoneurosome/nuclear microRNAs levels in control animals and 48 hours after SE.

	Control		Seizure	
MicroRNA Name	Syn/Nuc Ratio	T-test	Syn/Nuc Ratio	T-test
hsa-miR-923	**15.525**	**0.018**	0.918	0.885
ebv-miR-BART8*	**7.156**	**0.031**	0.840	0.788
hsa-miR-513a-5p	**6.474**	**0.002**	3.154	0.176
hsa-miR-371-5p	**5.365**	**0.015**	0.322	0.246
hsa-miR-498	**4.963**	**0.069**	0.410	0.326
miRPlus_28431	**4.434**	**0.006**	0.660	0.440
**mmu-miR-207/rno-miR-207**	**4.430**	**0.008**	1.481	0.207
**hsa-miR-494/mmu-miR-494/rno-miR-494**	**4.383**	**0.000**	1.270	0.551
**hsa-miR-933**	**4.021**	**0.001**	1.233	0.454
hsa-miR-520d-5p	**3.992**	**0.000**	0.830	0.591
hsa-miR-193a-5p	**3.968**	**0.011**	0.334	0.288
hsa-miR-185*	**3.853**	**0.000**	0.487	0.317
**hsa-miR-491-3p**	**3.834**	**0.010**	1.429	0.166
**mmu-miR-667**	**3.711**	**0.000**	1.132	0.576
hsa-miR-888*	**3.681**	**0.001**	1.010	0.957
*hsa-miR-519d	**3.510**	**0.001**	1.173	0.437
hsa-miR-658	**3.414**	**0.056**	0.845	0.762
mmu-miR-376b/rno-miR-376b-3p	**3.412**	**0.012**	0.717	0.293
mmu-miR-351/rno-miR-351	**3.363**	**0.000**	0.635	0.321
miRPlus_17952	**3.311**	**0.005**	0.251	0.242
mmu-miR-720	**3.291**	**0.001**	0.959	0.857
hsa-miR-149*	3.254	0.069	0.343	0.269
**hsa-miR-487b/mmu-miR-487b/rno-miR-487b**	**3.214**	**0.001**	0.917	0.666
mmu-miR-290-5p/rno-miR-290	**3.178**	**0.000**	0.512	0.317
hsa-miR-30c-2*/mmu-miR-30c-2*/rno-miR-30c-2*	**3.109**	**0.006**	0.875	0.754
mmu-miR-433*	**3.052**	**0.008**	1.139	0.622
**mmu-miR-434-3p/rno-miR-434**	**3.015**	**0.000**	0.840	0.465
hsa-miR-136*/rno-miR-136*	**3.012**	**0.000**	0.750	0.160
miRPlus_42487	**2.956**	**0.003**	0.227	0.212
hsa-miR-129*	**2.874**	**0.001**	0.868	0.563
rno-miR-204*	**2.872**	**0.001**	0.411	0.198
mmu-miR-376a/rno-miR-376a	**2.861**	**0.001**	**0.590**	**0.028**
**hsa-miR-551b/mmu-miR-551b/rno-miR-551b**	**2.859**	**0.000**	1.022	0.958
mmu-miR-675-5p	**2.853**	**0.018**	0.314	0.215
**hsa-miR-9/mmu-miR-9/rno-miR-9**	**2.834**	**0.004**	0.687	0.202
miRPlus_42793	**2.822**	**0.000**	0.593	0.190
hsa-miR-153/mmu-miR-153/rno-miR-153	**2.798**	**0.001**	0.657	0.072
hsa-miR-369-3p/mmu-miR-369-3p/rno-miR-369-3p	**2.781**	**0.000**	0.895	0.550
**hsa-miR-887**	**2.746**	**0.048**	1.356	0.143
hsa-miR-128/mmu-miR-128/rno-miR-128	**2.742**	**0.001**	0.734	0.226
**mmu-miR-384-3p/rno-miR-384-3p**	**2.742**	**0.000**	0.637	0.059
hsa-miR-335/mmu-miR-335-5p/rno-miR-335	**2.734**	**0.001**	0.808	0.285
**mmu-miR-300/rno-miR-300-3p**	**2.724**	**0.006**	0.849	0.554
**hsa-miR-34b/mmu-miR-34b-3p**	**2.705**	**0.012**	0.885	0.582
**hsa-miR-29c/mmu-miR-29c/rno-miR-29c**	**2.701**	**0.000**	**0.569**	**0.037**
hsa-miR-376a	**2.674**	**0.001**	0.659	0.086
**hsa-miR-137/mmu-miR-137/rno-miR-137**	**2.672**	**0.002**	0.820	0.361
**hsa-miR-503**	**2.619**	**0.000**	0.879	0.618
hsa-miR-9*/mmu-miR-9*/rno-miR-9*	**2.607**	**0.004**	0.676	0.114
**hsa-miR-381/mmu-miR-381/rno-miR-381**	**2.595**	**0.000**	0.728	0.202
hsa-miR-138-1*/mmu-miR-138*/rno-miR-138*	**2.590**	**0.004**	0.829	0.401
hsa-miR-7/mmu-miR-7a/rno-miR-7a	**2.565**	**0.000**	0.748	0.189
**hsa-miR-222/mmu-miR-222/rno-miR-222**	**2.518**	**0.007**	0.715	0.201
**hsa-miR-29b/mmu-miR-29b/rno-miR-29b**	**2.513**	**0.001**	**0.504**	**0.020**
hsa-miR-135a/mmu-miR-135a/rno-miR-135a	**2.504**	**0.005**	**0.565**	**0.015**
**rno-miR-742**	**2.498**	**0.014**	0.987	0.949
**hsa-miR-22/mmu-miR-22/rno-miR-22**	**2.480**	**0.000**	**0.602**	**0.043**
hsa-miR-129-3p/mmu-miR-129-3p/rno-miR-129*	**2.444**	**0.001**	0.707	0.150
**hsa-miR-99b/mmu-miR-99b/rno-miR-99b**	**2.431**	**0.006**	0.675	0.120
**hsa-miR-127-3p/mmu-miR-127/rno-miR-127**	**2.425**	**0.001**	0.776	0.387
**hsa-miR-99a/mmu-miR-99a/rno-miR-99a**	**2.420**	**0.005**	0.595	0.070
mmu-miR-138/rno-miR-138	**2.414**	**0.001**	0.637	0.082
**hsa-miR-98/mmu-miR-98/rno-miR-98**	**2.414**	**0.001**	0.677	0.121
**mmu-miR-582-3p**	**2.406**	**0.033**	1.054	0.823
**hsa-miR-100/mmu-miR-100/rno-miR-100**	**2.398**	**0.013**	**0.540**	**0.036**
hsa-miR-129-5p/mmu-miR-129-5p/rno-miR-129	**2.394**	**0.001**	0.710	0.194
hsa-miR-132/mmu-miR-132/rno-miR-132	**2.380**	**0.011**	0.743	0.195
hsa-miR-136/mmu-miR-136/rno-miR-136	**2.364**	**0.000**	**0.546**	**0.022**
**hsa-miR-138/mmu-miR-138/rno-miR-138**	**2.349**	**0.001**	**0.546**	**0.027**
**hsa-miR-125a-5p/mmu-miR-125a-5p/rno-miR-125a-5p**	**2.337**	**0.002**	0.689	0.182
**hsa-miR-124/mmu-miR-124/rno-miR-124**	**2.332**	**0.001**	**0.593**	**0.050**
hsa-miR-29a/mmu-miR-29a/rno-miR-29a	**2.302**	**0.001**	**0.499**	**0.017**
**hsa-miR-125b/mmu-miR-125b-5p/rno-miR-125b-5p**	**2.299**	**0.002**	0.633	0.087
**hsa-miR-221/mmu-miR-221/rno-miR-221**	**2.297**	**0.002**	0.731	0.196
**hsa-miR-30c/mmu-miR-30c/rno-miR-30c**	**2.274**	**0.006**	**0.579**	**0.044**
rno-miR-135a*	**2.259**	**0.005**	0.831	0.629
**hsa-miR-361-5p/mmu-miR-361/rno-miR-361**	**2.230**	**0.001**	0.751	0.234
mmu-miR-872*/rno-miR-872*	**2.207**	**0.010**	0.759	0.150
hsa-miR-185/mmu-miR-185/rno-miR-185	**2.189**	**0.000**	**0.604**	**0.031**
**hsa-miR-218/mmu-miR-218/rno-miR-218**	**2.189**	**0.001**	**0.504**	**0.013**
mghv-miR-M1-4	**2.188**	**0.001**	0.494	0.202
**hsa-miR-103/mmu-miR-103/rno-miR-103**	**2.185**	**0.001**	0.794	0.269
**hsa-let-7d/mmu-let-7d/rno-let-7d**	**2.183**	**0.000**	**0.573**	**0.038**
miRPlus_17869	**2.173**	**0.000**	0.270	0.188
**hsa-miR-411/mmu-miR-411/rno-miR-411**	**2.165**	**0.001**	0.717	0.094
**hsa-miR-107/mmu-miR-107/rno-miR-107**	**2.161**	**0.006**	0.713	0.176
hsa-miR-637	2.160	0.225	0.853	0.760
**hsa-miR-30a/mmu-miR-30a/rno-miR-30a**	**2.153**	**0.003**	**0.574**	**0.042**
**hsa-miR-30e/mmu-miR-30e/rno-miR-30e**	**2.147**	**0.002**	**0.539**	**0.026**
mmu-miR-300*/rno-miR-300-5p	**2.140**	**0.015**	0.435	0.150
hsa-miR-30d/mmu-miR-30d/rno-miR-30d	**2.136**	**0.004**	0.709	0.138
**hsa-let-7b/mmu-let-7b/rno-let-7b**	**2.121**	**0.005**	**0.549**	**0.028**
hsa-miR-149/mmu-miR-149	**2.113**	**0.000**	0.819	0.264
**hsa-let-7a/mmu-let-7a/rno-let-7a**	**2.101**	**0.001**	**0.555**	**0.034**
**hsa-miR-30b/mmu-miR-30b/rno-miR-30b-5p**	**2.092**	**0.005**	**0.529**	**0.022**
**hsa-miR-130a/mmu-miR-130a/rno-miR-130a**	**2.086**	**0.003**	**0.589**	**0.022**
hsa-miR-516a-5p	2.077	0.107	0.297	0.222
**hsa-miR-26b/mmu-miR-26b/rno-miR-26b**	**2.065**	**0.003**	**0.533**	**0.019**
**hsa-miR-342-3p/mmu-miR-342-3p/rno-miR-342-3p**	**2.061**	**0.001**	0.799	0.381
**hsa-miR-634**	**2.057**	**0.000**	0.892	0.576
**rno-miR-352**	**2.045**	**0.001**	0.619	0.073
**mmu-let-7d/rno-let-7d**	**2.029**	**0.004**	**0.534**	**0.026**
mmu-miR-711	**2.027**	**0.000**	0.346	0.231
**hsa-miR-181a/mmu-miR-181a/rno-miR-181a**	**2.014**	**0.012**	0.707	0.163
ebv-miR-BART2-3p	**2.010**	**0.003**	0.734	0.429
**mmu-miR-101b/rno-miR-101b**	**2.008**	**0.022**	**0.574**	**0.045**
**hsa-miR-154/mmu-miR-154/rno-miR-154**	**2.007**	**0.001**	0.636	0.062
hsa-miR-921	**2.006**	**0.003**	0.650	0.296
**hsa-miR-191/mmu-miR-191/rno-miR-191**	**2.002**	**0.003**	0.662	0.091
**mmu-let-7g**	**1.981**	**0.012**	**0.471**	**0.010**
**hsa-let-7g/mmu-let-7g**	**1.966**	**0.002**	0.641	0.087
**hsa-miR-301a/mmu-miR-301a/rno-miR-301a**	**1.964**	**0.011**	**0.590**	**0.020**
**hsa-let-7f/mmu-let-7f/rno-let-7f**	**1.957**	**0.001**	0.631	0.076
**hsa-miR-26a/mmu-miR-26a/rno-miR-26a**	**1.955**	**0.001**	**0.532**	**0.020**
**mmu-miR-329/rno-miR-329**	**1.938**	**0.001**	0.668	0.085
hsa-miR-101/mmu-miR-101a/rno-miR-101a	**1.937**	**0.003**	**0.528**	**0.010**
**mmu-miR-325/rno-miR-325-3p**	**1.899**	**0.000**	0.746	0.195
hsa-miR-602	**1.891**	**0.044**	0.246	0.298
mmu-miR-503*	**1.847**	**0.002**	0.772	0.280
**mmu-let-7f/rno-let-7f**	**1.844**	**0.010**	**0.467**	**0.010**
hsa-miR-708/mmu-miR-708/rno-miR-708	**1.834**	**0.001**	0.689	0.094
hsa-miR-379/mmu-miR-379/rno-miR-379	**1.797**	**0.013**	**0.590**	**0.026**
**hsa-let-7c/mmu-let-7c/rno-let-7c**	**1.783**	**0.005**	**0.491**	**0.016**
mmu-miR-883b-5p	1.775	0.092	0.979	0.947
hsa-miR-140-3p/mmu-miR-140*/rno-miR-140*	**1.770**	**0.003**	0.595	**0.029**
**hsa-miR-744/mmu-miR-744**	**1.766**	**0.024**	0.673	0.115
**hsa-miR-382/mmu-miR-382/rno-miR-382**	**1.755**	**0.016**	0.917	0.693
miRPlus_27560	1.748	0.248	0.341	0.279
hsa-miR-30e*/mmu-miR-30e*/rno-miR-30e*	**1.747**	**0.013**	0.737	0.168
mmu-miR-483	**1.679**	**0.002**	0.551	0.239
**hsa-miR-195/mmu-miR-195/rno-miR-195**	**1.634**	**0.019**	**0.463**	**0.005**
hsa-miR-23a/mmu-miR-23a/rno-miR-23a	**1.628**	**0.019**	**0.435**	**0.005**
hsa-miR-142-5p/mmu-miR-142-5p/rno-miR-142-5p	1.613	0.308	0.437	0.051
hsa-miR-16/mmu-miR-16/rno-miR-16	**1.607**	**0.029**	**0.402**	**0.007**
**mmu-let-7i/rno-let-7i**	**1.607**	**0.027**	**0.382**	**0.002**
**hsa-miR-181b/mmu-miR-181b/rno-miR-181b**	**1.607**	**0.027**	0.735	0.190
**hsa-miR-340/mmu-miR-340-5p/rno-miR-340-5p**	**1.587**	**0.011**	**0.546**	**0.017**
hsa-miR-34a/mmu-miR-34a/rno-miR-34a	**1.582**	**0.002**	**0.629**	**0.012**
**hsa-miR-185/mmu-miR-185/rno-miR-185**	**1.578**	**0.003**	**0.432**	**0.036**
hsa-miR-193b*	1.522	0.083	0.331	0.274
hsa-miR-23b/mmu-miR-23b/rno-miR-23b	**1.508**	**0.017**	**0.412**	**0.005**
**hsa-miR-146b-5p/mmu-miR-146b/rno-miR-146b**	**1.507**	**0.038**	**0.625**	**0.013**
**hsa-let-7i/mmu-let-7i/rno-let-7i**	**1.499**	**0.032**	**0.350**	**0.002**
hsa-miR-30b*	**1.487**	**0.026**	0.369	0.202
hsa-miR-24/mmu-miR-24/rno-miR-24	**1.480**	**0.031**	**0.372**	**0.006**
hsa-miR-320/mmu-miR-320/rno-miR-320	1.447	0.188	0.680	0.061
**miRPlus_42856**	1.424	0.164	**0.458**	**0.036**
hsa-miR-15a/mmu-miR-15a	**1.399**	**0.030**	**0.359**	**0.009**
mmu-miR-467e*	**1.389**	**0.041**	0.590	0.156
hsa-miR-20a/mmu-miR-20a/rno-miR-20a	**1.371**	**0.012**	**0.526**	**0.021**
hsa-miR-338-3p/mmu-miR-338-3p/rno-miR-338	**1.355**	**0.047**	**0.396**	**0.016**
hsa-miR-424	1.354	0.329	**0.437**	**0.016**
hsa-miR-19b/mmu-miR-19b/rno-miR-19b	1.339	0.077	**0.459**	**0.019**
hcmv-miR-UL148D	**1.322**	**0.002**	0.821	0.221
mmu-miR-483*/rno-miR-483	**1.308**	**0.000**	0.827	0.232
hsa-miR-483-3p	**1.308**	**0.042**	0.834	0.337
mmu-miR-106a	**1.295**	**0.044**	**0.543**	**0.014**
hsa-miR-765	1.238	0.265	0.358	0.095
hsa-miR-106a	1.215	0.275	0.553	**0.019**
hsa-miR-204/mmu-miR-204/rno-miR-204	1.175	0.733	0.657	0.086
hsa-miR-142-3p/mmu-miR-142-3p/rno-miR-142-3p	1.156	0.651	**0.291**	**0.033**
**rno-miR-664**	1.133	0.308	**0.597**	**0.030**
hsa-miR-17/mmu-miR-17/rno-miR-17-5p/rno-miR-17	1.128	0.461	**0.578**	**0.037**
hsa-miR-300	1.121	0.444	0.196	0.080
hsa-miR-27b/mmu-miR-27b/rno-miR-27b	1.119	0.339	**0.303**	**0.006**
**hsa-miR-574-3p/mmu-miR-574-3p**	1.105	0.329	**0.489**	**0.024**
**rno-miR-450a**	1.065	0.785	0.319	0.326
hsa-miR-21/mmu-miR-21/rno-miR-21	1.038	0.566	0.409	0.082
hsa-miR-518c*	0.992	0.957	0.348	0.122
hsa-miR-15b/mmu-miR-15b/rno-miR-15b	0.975	0.908	**0.450**	**0.023**
hsa-miR-583	0.951	0.772	0.329	0.147
hsa-miR-219-2-3p/rno-miR-219-2-3p	0.948	0.762	**0.431**	**0.039**
kshv-miR-K12-8	0.947	0.786	0.292	0.068
hsa-miR-27a/mmu-miR-27a/rno-miR-27a	0.933	0.560	**0.243**	**0.009**
mmu-miR-466f-3p	0.899	0.717	**0.265**	**0.005**
hsa-miR-219-5p/mmu-miR-219/rno-miR-219-5p	0.893	0.421	**0.231**	**0.014**
**hsa-miR-150/mmu-miR-150/rno-miR-150**	0.860	0.245	0.584	0.118
**hsa-miR-378/mmu-miR-378/rno-miR-378**	0.842	0.259	**0.475**	**0.001**
hsa_SNORD3@	0.840	0.208	0.245	0.073
hsa-miR-34c-5p/mmu-miR-34c/rno-miR-34c	0.827	0.619	**0.498**	**0.000**
mmu-miR-297b-3p/mmu-miR-297a*/mmu-miR-297c*	0.789	0.131	**0.293**	**0.001**
hsa-miR-485-3p/mmu-miR-485*	0.770	0.077	**0.383**	**0.040**
hsa-miR-642	0.764	0.063	0.295	0.080
hsa-miR-620	0.722	0.116	0.341	**0.006**
mmu-miR-883a-5p	0.720	0.130	0.260	0.050
mmu-miR-882	0.718	0.053	0.196	0.080
hsa-miR-423-5p/mmu-miR-423-5p	**0.714**	**0.043**	0.376	0.056
hsa-let-7e/mmu-let-7e/rno-let-7e	**0.700**	**0.013**	**0.198**	**0.001**
mmu-miR-685	0.670	0.630	0.252	0.158
hsa-miR-223/mmu-miR-223/rno-miR-223	0.666	0.117	0.383	0.073
mmu-miR-34b-5p/rno-miR-34b	0.659	0.495	**0.432**	**0.003**
hsa-miR-550	**0.566**	**0.020**	**0.234**	**0.002**
hsa-miR-620	0.544	0.058	**0.487**	**0.011**
hsa-miR-574-5p/mmu-miR-574-5p	**0.535**	**0.003**	**0.269**	**0.005**
**ebv-miR-BART17-5p**	**0.527**	**0.016**	**0.247**	**0.013**
hsa-miR-519c-5p/hsa-miR-519b-5p/hsa-miR-523*/hsa-miR-518e*/hsa-miR-522*/hsa-miR-519a*	**0.515**	**0.016**	0.316	0.070
mmu-miR-881*	**0.484**	**0.009**	0.182	0.052
mmu-miR-466b-3-3p	**0.481**	**0.013**	**0.366**	**0.032**
mmu-miR-467a*/mmu-miR-467d*	**0.473**	**0.006**	**0.229**	**0.005**
mmu-miR-466d-3p	**0.445**	**0.013**	**0.291**	**0.001**
mmu-miR-467b*	**0.441**	**0.002**	**0.230**	**0.003**
hsa-miR-659	**0.435**	**0.001**	**0.220**	**0.017**
**mmu-miR-327**	0.421	0.077	**0.160**	**0.001**
mmu-miR-468	**0.419**	**0.001**	**0.295**	**0.015**
rno-miR-466c	**0.416**	**0.017**	**0.243**	**0.007**
**miRPlus_28302**	**0.408**	**0.010**	**0.125**	**0.019**
hsa-miR-801/mmu-miR-801	**0.403**	**0.001**	**0.154**	**0.002**
hsa-miR-549	**0.393**	**0.001**	**0.144**	**0.005**
hsa-miR-32*	**0.385**	**0.013**	**0.201**	**0.018**
rno-miR-466b	**0.363**	**0.002**	**0.276**	**0.006**
mmu-miR-297c	**0.358**	**0.027**	**0.311**	**0.017**
mmu-miR-466b-5p	**0.358**	**0.011**	**0.221**	**0.015**
mmu-miR-466a-5p	**0.357**	**0.003**	**0.231**	**0.012**
**hsa-miR-298**	**0.353**	**0.000**	**0.178**	**0.011**
hsa_SNORD2	**0.337**	**0.000**	**0.147**	**0.006**
mmu-miR-466e-5p	**0.298**	**0.002**	**0.284**	**0.011**
hsa-miR-936	**0.285**	**0.003**	**0.097**	**0.014**
mmu-miR-466d-5p	**0.279**	**0.014**	**0.227**	**0.011**
hsa-miR-939	**0.267**	**0.002**	**0.170**	**0.006**
hsa-miR-451/mmu-miR-451/rno-miR-451	**0.258**	**0.022**	0.065	0.095
mmu-miR-466c-5p	**0.251**	**0.009**	**0.219**	**0.010**
hsa-miR-126*/mmu-miR-126-5p/rno-miR-126*	**0.192**	**0.000**	**0.074**	**0.011**
**U6-snRNA-1**	**0.157**	**0.006**	**0.109**	**0.002**
mmu-miR-690	**0.152**	**0.005**	**0.077**	**0.002**
**mmu-miR-875-3p**	**0.150**	**0.002**	**0.123**	**0.005**
**hsa-miR-145/mmu-miR-145/rno-miR-145**	**0.116**	**0.000**	**0.109**	**0.002**
**mmu-miR-691**	**0.116**	**0.003**	**0.065**	**0.001**
U6-snRNA-2	**0.105**	**0.001**	**0.050**	**0.004**
hsa-miR-526b*	**0.096**	**0.000**	**0.168**	**0.001**
**hsa-miR-665**	**0.090**	**0.000**	**0.058**	**0.003**
**hsa-miR-126/mmu-miR-126-3p/rno-miR-126**	**0.070**	**0.000**	**0.040**	**0.007**
hsa-miR-143/mmu-miR-143/rno-miR-143	**0.053**	**0.000**	**0.033**	**0.001**
hsa-miR-302d*	**0.049**	**0.004**	**0.036**	**0.000**
mmu-miR-709	**0.031**	**0.001**	**0.021**	**0.001**

Syn-synaptoneurosomes, Nuc-nuclear; Ratios bolded if >1, or less than <1 and have a t-test P value<0.05. MicroRNA names bolded were decreased in whole hippocampal arrays.

The microRNAs shown in red on Figure 3b have previously been reported as dendritic or synaptoneurosome enriched. [10], [11], [32]. The microRNAs shown in green on Figure 3b have been previously reported to have low levels of expression in dendrites or synaptoneurosomes [10], [11], [32]. MicroRNA expression in the hippocampal synaptoneurosomes and nuclear fractions from the control animals were mostly consistent with the findings in prior studies.

Of the microRNAs present on the synaptoneurosome/nuclear fraction arrays, 100 were also microRNAs that were identified as decreased in the whole hippocampal sample 48 hours post SE. These are bolded and highlighted in yellow on Table 4. Eighty-four of the 100 were synaptoneurosome enriched in the control hippocampus, synaptoneurosome/nuclear >1. Five were equally distributed between the synaptoneurosome and nuclear fractions, synaptoneurosome/nuclear ∼1. Eleven were nuclear enriched, synaptoneurosome/nuclear<1.

**Figure 4:**
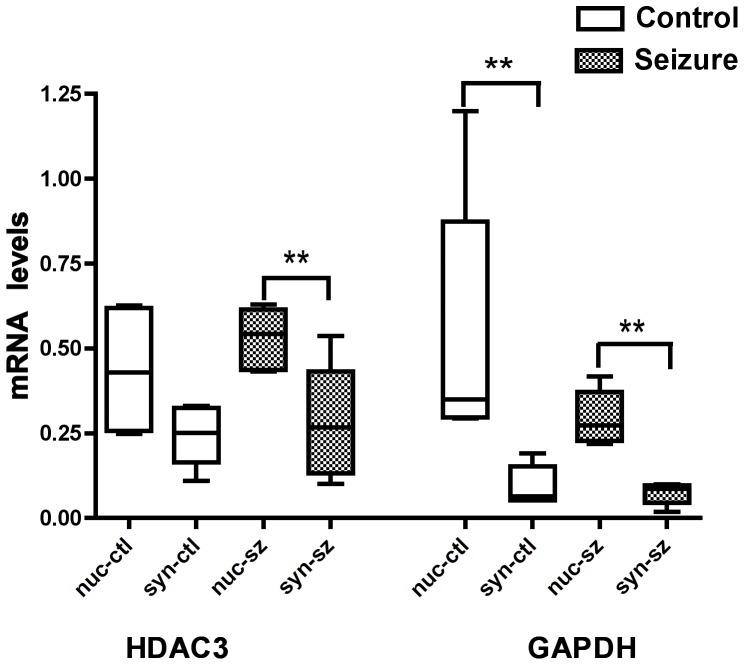
Real-time PCR demonstrated that HDAC3 and GAPDH mRNAs are enriched in the nuclear compared to synaptoneurosome fractions of both control and SE treated animal’s hippocampi. N = 6 for all samples, Nuclear control (nuc-ctl), synaptoneurosome control (syn-ctl), Nuclear SE (nuc-sz), synaptoneurosome SE (syn-sz), P**<0.01 Mann-Whitney.

To assess our ability to fractionate nuclear and synaptoneurosome RNA we used real time PCR to measure two mRNAs that had previously been shown to be enriched in the nuclear fraction, histone deacetylase three (HDAC3) and glyceraldehyde-3-phosphate dehydrogenase (GAPDH) [33]. GAPDH mRNA was enriched in the nuclear fraction from the hippocampus of the control and the SE treated animals. (Figure 4, N = 6 for all groups; control nuclear-0.5±0.14, synaptoneurosome-0.09±0.02; SE nuclear-0.3±0.03, synaptoneurosome-0.08±0.03, mean±SEM, **P<0.01 Mann-Whitney). HDAC3 mRNA was not enriched in the nuclear fraction of the control animals (Figure 4, N = 6 for all groups; control nuclear-0.44±0.07, synaptoneurosome-0.24±0.03, mean±SEM, P = 0.06 Mann-Whitney). HDAC3 mRNA was enriched in the nuclear fraction of the SE animals (Figure 4, N = 6 for all groups; SE nuclear-0.53±0.03, synaptoneurosome-0.28±0.06, mean±SEM, **P<0.01 Mann-Whitney) Forty-eight hours after SE, HDAC3 and GAPDH mRNA levels did not change significantly in the nuclear or synaptoneurosome fractions.

**Figure 5:**
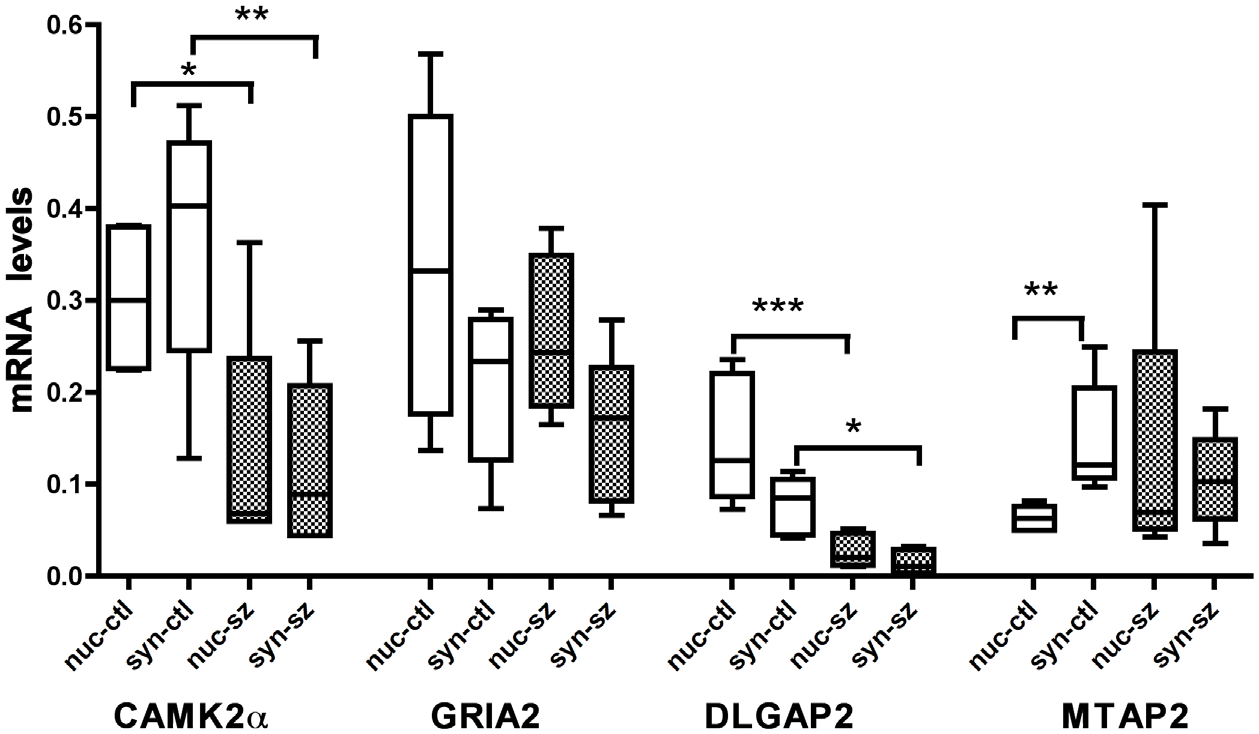
Real-time PCR demonstrated that MTAP3 mRNA is enriched in the synaptoneurosome compared to nuclear fractions of control animals hippocampus, Mann-Whitney P**<0.01. MTAP3 mRNA levels are similar in synaptoneurosome and nuclear fractions 48 hours after SE. CAMK2α, GRIA2 and DLGAP2 mRNA as measured by real-time PCR was similar in the synaptoneurosome and nuclear fractions of both control and SE animal’s hippocampi. Following SE CAMK2α and DLGAP2 mRNA decreased in both the nuclear and synaptoneurosome fractions, P * 0.05, P** 0.01, P*** 0.001 ANOVA with Bonferroni corrections N = 6 for all samples, nuclear control (nuc-ctl), synaptoneurosome control (syn-ctl), nuclear SE (nuc-sz), synaptoneurosome SE (syn-sz).

We next used real time PCR to measure the levels in the nuclear and synaptoneurosome fractions of 4 mRNAs previously identified as expressed in dendrites: Ca++/calmodulin dependant protein kinase IIα (CAMK2α), GLUR2 subunit of the α-amino-3hydroxy-5-methyl-4-isocazolepropionic acid (AMPA) receptor (GRIA2), post-synaptic density gaunylate kinase (DLGAP2), and microtubule associated protein 2 (MTAP2) (Figure 5). All 4 mRNAs were present in the synaptoneurosome fraction and three were present in equal abundance in the synaptoneurosome and nuclear fractions, only MTAP2 was enriched in the synaptoneurosome fraction (N = 6 for all groups, control nuclear-0.06±0.01 and synaptoneurosome-0.14±0.02, mean±SEM P***<0.01 Mann-Whitney). The data suggest that HDAC3 and GAPDH mRNAs are nuclear enriched and while the four mRNAs (CAMK2α, GRIA2, DLGAP2, MTAP2) are highly expressed in the synaptoneurosome fraction, only MTAP2 mRNA was enriched.

To determine if there is a decrease in the synaptoneurosome/nuclear ratio of mRNAs 48 hours after SE, similar to the reduction in microRNAs; CAMK2α, GRIA2, DLGAP2, and MTAP2 mRNA levels were compared in the synaptoneurosome and nuclear fractions of controls and SE treated animals. CAMK2α and DLGAP2 mRNA levels decreased in both the nuclear and synaptoneurosome fractions 48 hours after SE (N = 6 for all groups, CAMK2α: control nuclear-0.30±0.03, SE nuclear −0.12±0.05, mean±SEM, P*<0.05 ANOVA with Bonferroni corrections; control synaptoneurosome −0.37±0.05 mean±SEM, SE synaptoneurosome −0.11±0.08 mean±SEM P**<0.01 ANOVA with Bonferroni multiple corrections; DLGAP2: control nuclear fraction −0.15±0.03, mean±SEM, SE nuclear fraction 0.03±0.01, mean±SEM, P***<0.0001 ANOVA with Bonferroni corrections; control synaptoneurosome fraction −0.08±0.01 mean±SEM, SE synaptoneurosome fraction −0.01±0.004 mean±SEM P*<0.05 ANOVA with Bonferroni multiple corrections). The MTAP2 mRNA enrichment in synaptoneurosomes seen in controls was not present following SE. This was at least partially due to increased MTAP2 mRNA in the nuclear sample following SE (N = 6 for all groups mean±SEM, Control nuclear −0.063±0.01, Control synaptoneurosome −0.144±0.02, SE nuclear −0.13±0.06, SE synaptoneurosome −0.10±0.02). GRIA2 mRNA levels were unchanged in both the nuclear and synaptoneurosome fractions following SE. Following SE the synaptoneurosome/nuclear ratio of CAMK2α, GRIA2 and DLGAP2 mRNAs did not change. There was, however, a decrease in the synaptoneurosome/nuclear ratio of MTAP2 mRNA but this was accompanied by an increase in nuclear MTAP2 mRNA levels 48 hours after SE.

## Discussion

Here we show that following SE a subset of microRNAs increase at 4 hours, 48 hours and 3 weeks following SE. At the first time point we studied, 4 hours post-SE no microRNAs decreased. Forty-eight hours after SE however, 188 microRNAs decreased and by 3 weeks after SE only three microRNAs had decreased. The very specific decrease in 188 microRNAs only at 48 hours after SE suggests either a simultaneous decrease in production or increased degradation of a large number of microRNAs at a specific period of time following SE.

To begin to understand the mechanism underlying the decrease in microRNAs at 48 hours we carried out subcellular fractionation of the hippocampus and measured the synaptoneurosome/nuclear ratio of microRNAs following SE. Similar to prior studies we found a large pool of microRNAs enriched in the synaptoneurosome fraction compared to the nuclear fraction of the control hippocampus [10]. Forty-eight hours after SE there were almost no synaptoneurosome enriched microRNAs. The decrease in microRNAs in the whole hippocampal sample correlates with a decrease in the synaptoneurosome/nuclear microRNA ratio. The decrease in the synaptoneurosome/nuclear ratio 48 hours after SE would be consistent with either a reduction in microRNA levels in the synaptoneurosomes, a shift of microRNAs from the synaptoneurosome to nuclear fraction or an increased microRNA levels in the nuclear fraction. While a shift of microRNAs from the synaptoneurosome to the nuclear fraction or an increase in microRNA in the nuclear fraction could explain the reduction in the synaptoneurosome/nuclear ratio it would be inconsistent with the large number of microRNAs that decreased at 48 hours in the whole hippocampal samples.

MicroRNA expression can be regulated at multiple steps including transcription, post-transcriptional processing and degradation of mature microRNAs [34], [35]. The half-lives of mature microRNAs vary greatly over minutes to weeks with most being days to weeks [36]. In culture altering the cellular density can shorten the half-lives of some microRNAs from days to less than an hour [37]. Neuronal activity *in vitro* and *in vivo* can trigger increased degradation of microRNAs [35]. Our finding of multiple decreased microRNAs at 48 hours after SE would be consistent with a shorter half-life for some microRNAs after SE, but future studies *in vivo* would be needed to confirm this finding. Taken together our current hypothesis is that decreases in the overall levels of microRNAs 48 hours after SE are related to a reduction in synaptoneurosome microRNAs due to increased degradation. Future studies are being directed at tagging microRNAs to measure processing, subcellular targeting and degradation following SE to better understand the mechanism of microRNA loss 48 hours after SE.

We used a low threshold for identifying changes in microRNAs following SE on the arrays, t-test less than 0.05. We were able to validate changes in 6 of 8 microRNAs using real time PCR at 48 hours after SE. Suggesting that many but certainly not all of the microRNA changes we identified on the arrays following SE are independent of the technique.

Prior studies in epilepsy models have identified a variety of microRNAs that change following SE [4], [5], [6], [38], [39]. None of them, however, used an identical epilepsy model, microRNA measurement platforms or time points as the present study making cross study comparisons difficult. All of the array studies identified a subset of microRNAs that increased and a subset that decreased. Only a small subset of the microRNAs they identified as changing after seizures also changed in our study and the four previously published studies had few microRNA changes in common. Four of the five studies, including ours, identified an increase in miR132 [32]. Three studies, including ours identified an increase in mir21, and a decrease in miR98. No other microRNA changes were identified in more than two studies, suggesting a great deal of heterogeneity and time course specific changes in microRNAs following a seizure.

The finding of an abundance of microRNAs enriched in the synaptoneurosome of the hippocampus is similar to a prior study of synaptoneurosomes from mouse forebrain [10]. Of the 20 most highly synaptoneurosome enriched microRNAs in the Lugli et al. 2008 paper four were also enriched in our hippocampal synaptoneurosomes (shown in red on Figure 3). Of the 10 microRNAs enriched in the nuclear fraction in their study five were nuclear enriched in our study (shown in green on Figure 3B) and three trended toward nuclear enrichment. Another study using laser capture to isolate dendrites and nuclear samples from hippocampal cultures identified five microRNAs that were in greater concentrations in the dendrites than cell bodies, of these two were enriched in our synaptoneurosome fraction (shown in red on Figure 3b) [11]. A more recent study using array and confirmed with real-time PCR identified three microRNAsas synaptoneurosome and three nuclear enriched in the cortex. All six microRNAs were similarly enriched in our hippocampal samples from control animals. There is a large pool of microRNAs present in the synaptoneurosome fraction and a subset appears to be highly enriched.

A prolonged seizure causes swelling, blebbing and loss of normal dendritic architecture [19], [40]. The mechanism of this dendritic damage is not clear though the changes are most severe during the seizure with some recovery post seizure. In the present study the rats had strong seizure activity, stage III-V Racine scale, for an hour prior to receiving valium. While none of these animals underwent VEEG recording it is likely the seizure activity was subsiding by 4 hours. By 48 hours they look relatively well and if having seizure they showed minimal behavioral signs. If the decrease we see in microRNAs at 48 hours were due to a direct loss of synaptic boutons that probably would have been most severe at 4 hours post SE. Instead we suspect, though do not prove, that there was an increase in the rates of microRNA degradation in the synaptoneurosome fraction following SE.

The lack of a change in the synaptoneursome/nuclear ratio of CAMK2α, GRIA2, and DLGAP2, 48 hours after SE suggests that the change in microRNAs synaptoneursome/nuclear ratio might be specific to microRNAs and not due to a global effect on RNA. To prove this further studies focused on the mechanism of the microRNA loss are needed.

Previous studies found a specific loss of GRIA2 mRNA in the CA3 pyramidal cell layer which we did not find in the fractionated whole hippocampus [41]. The decrease in CAMK2α mRNA in both the nuclear and synaptoneurosome fractions following SE may be contributing to the development of epilepsy as transgenic mice lacking CAMK2α are epileptic [42]. Prior studies *in vitro* and *in vivo* suggest that CAMK2α activity is down regulated in multiple seizure models, however, not due to a decrease in the level of the CAMK2α protein [43], [44], [45]. Further studies to understand the cause of the decrease in CAMK2α mRNA, its effect on CAMK2α activity and how it contributes to epileptogenesis may be warranted.

Local mRNA translation in dendrites plays an important role in the regulation of synapse structures and functions. Activity dependent regulation of synapse stability via mRNA translation in dendrites appears to be partially mediated by a subset of microRNAs [17], [46], [47]. Our finding that an episode of SE causes a decrease in synaptoneurosome microRNAs suggests that SE could disrupt the ability of synapses to respond to normal activity cues. Future studies to better understand the mechanism and the implications for loss of synaptoneurosome microRNAs following SE will have implications for epileptogenesis and learning and memory impairment in epilepsy.
